# Wide-Angle, Ultra-Wideband ISAR Imaging of Vehicles and Drones

**DOI:** 10.3390/s18103311

**Published:** 2018-10-02

**Authors:** Chenchen J. Li, Hao Ling

**Affiliations:** 1 Southwest Research Institute, San Antonio, TX 78238, USA; 2 Department of Electrical & Computer Engineering, The University of Texas at Austin, Austin, TX 78712, USA; ling@ece.utexas.edu

**Keywords:** radar imaging, inverse synthetic aperture radar, radar measurements, ultra-wideband, vehicles, drones

## Abstract

In-situ, wide-angle, and ultra-wideband inverse synthetic aperture radar (ISAR) imaging of vehicles and drones is demonstrated using a portable ultra-wideband radar. In order to form well-focused ISAR images, motion compensation is performed before applying the *k*-space imaging algorithm. While the same basic motion compensation methodology is applied to both types of targets, a more complex motion model is needed to better capture the flight path of the drone. The resulting ISAR images clearly show the geometrical outline of the targets and highlight locations of prominent backscattering. The ISAR images are also assessed against images generated through instrumented targets or laboratory measurements, and the image quality is shown to be comparable.

## 1. Introduction

Inverse synthetic aperture radar (ISAR) imaging is a standard radar mode for target identification. The typical ISAR imaging scenario consists of a stationary radar and a maneuvering target, where different aspects of target reflectivity are collected through target motion. The range and cross-range resolutions are inversely proportional to the bandwidth of the radar and the aspect swath of the target from target rotation, respectively. A key challenge of ISAR is that blind motion compensation is needed to form a focused image since the motion of the target is generally not known. Sophisticated motion compensation algorithms must be applied to arrive at a focused image [1,2,3,4,5,6,7,8]. Usually, these algorithms are based on some motion model (for both translation and rotation) with unknown parameters. The assumption that one or more prominent point scatterers on the target are visible throughout the image collection interval is then made to determine the motion parameters from the data and generate a focused image.

While radar-imaging techniques (both SAR and ISAR) are well established, recent advances in radio-frequency integrated circuits have significantly reduced the size and weight of radar components, expanding radar imaging beyond purely military applications. One particularly interesting area is the development of commercial ultra-wideband (UWB) technology. In 2002, the United States Federal Communications Commission (FCC) allocated spectrum for the commercial usage of low-power UWB technologies for communications and sensing [9]. Since then, commercial UWB devices have become popular. One of the great appeals of UWB devices for radar applications is that they can provide high-resolution range information (at the centimeter level) due to the wide bandwidth. The design of receiver architecture has been advanced to deal with the wide instantaneous bandwidth [10,11,12]. A low-power realization is implemented by the Time Domain Corporation in their commercial UWB transceivers. The latest generation is the PulsON 400 series [13]. Its transmitter emits short pulses at a pulse repetition frequency (PRF) of 10 MHz, with an equivalent frequency bandwidth from 3.1 to 5.3 GHz. The pulse shape is engineered to be compatible with FCC regulations. Furthermore, the pulses are pseudo-random coded to overcome the 15 m maximum unambiguous range (due to the 10 MHz PRF) and improve the signal-to-noise ratio (SNR). The transceiver unit provides a low-cost platform with low power consumption, small form factor (contained on a single 3” by 3.15” board), and a convenient universal serial bus (USB) interface. It opens many new possibilities of portable UWB radar measurements, which were prohibitive in the past.

Because of their large pulse bandwidth, UWB transceivers can provide high-resolution range information. However, in order to obtain equally high resolution in cross-range, the physical or synthetic collection aperture must also be increased. For physical apertures, the use of 1-D arrays combined with UWB radars has been investigated in the context of through-wall imaging [14,15,16,17,18,19]. Advanced signal processing algorithms such as MUSIC or RELAX have also be applied to further reduce the physical size of antenna arrays [20,21,22]. For synthetic aperture imaging, some previous works on generating high-resolution SAR [19,23] and ISAR images [24] have been reported in the literature. However, they are laboratory measurements, where the synthetic collection aperture is increased on well-controlled rails or turntables. The use of UWB radars for in-situ synthetic aperture collection warrants further investigation.

In this paper, we investigate and demonstrate the use of a portable UWB radar system to achieve in-situ, high-resolution radar imaging of moving targets. The scientific question we set out to address is the following. Since UWB radar provides high range resolution, can we achieve an equally high cross-range resolution through an increased synthetic aperture and motion compensation? Only if this is realizable will a high-resolution, two-dimensional image result, thus fulfilling the true potential of UWB imaging. In the wide collection aperture scenario, a target can deviate significantly from the highly idealized motions assumed in the motion compensation model, making the motion compensation task much more challenging. Moreover, it may not be possible to find a persistent point scatterer that is visible over the entire imaging window. ISAR imaging of two classes of moving targets, vehicles and small drones, are reported in this work. We develop measurement methodologies, collect data using UWB transceivers from Time Domain, and implement motion compensation and image formation algorithms to form high-resolution radar images.

This paper is organized as follows. In Section 2, Section 3 and Section 4, we discuss the ISAR imaging of vehicles. We investigate whether ISAR imaging of moving vehicles from a stationary radar may also be realized under proper conditions. In Section 5, Section 6 and Section 7, we discuss the ISAR imaging of a small drone. We investigate whether the vehicle imaging methodology can be extended to image a drone in flight. Finally, conclusions are given in Section 8.

## 2. Vehicle Imaging and Measurement Setup

Radar is a possible alternative to optical cameras for vehicle sensing in traffic monitoring, perimeter security, and autonomous driving applications [25,26,27,28,29,30,31,32,33]. SAR imaging of stationary ground vehicles from airborne sensors has already been a subject of extensive investigations in the past [25,26,27,28]. ISAR imaging of moving vehicles from a stationary ground radar has also been realized in the small-angle scenario previously [31,32]. Wide-angle imaging may offer better resolution and richer geometrical features. In Reference [33], 360° ISAR imaging was carried out to obtain a high-resolution image map of a vehicle. However, the measurement was done on a turntable. In Reference [34], we reported on the cooperative wide-angle UWB imaging of vehicles, where the imaged vehicles are instrumented to provide range information. In this paper, we address the non-cooperative scenario, where the vehicle range is not known and will need to be estimated from the measured range profile data. To collect the data, the PulsON 410 (P410) UWB transceiver is used as a mono-static radar [13]. The sampling rate for consecutive range profiles is chosen such that: (i) It is greater than the Doppler extent of the target to ensure no Doppler aliasing occurs, and (ii) it is as low as possible to maximize the number of coherent pulse integration to reduce noise. In this case, it is set to 67 Hz. A single dual-ridged broadband horn (Dorado International, GH1-12N, 1–12 GHz) is employed for both transmit and receive, through the use of a circulator (CS-16-50 from MLCI Corporation). Vertical polarization is used on transmit and receive. Note that the 10 MHz pulse repetition frequency is fixed in the firmware of the radar and is not adjustable by the user. Table 1 summarizes the measurement parameters.

**Figure 1:**
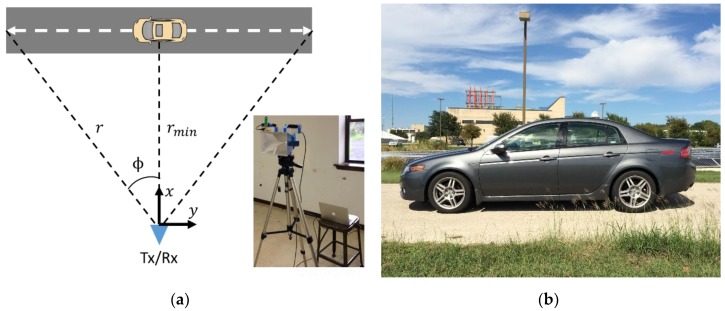
Vehicle measurement setup. (**a**) Measurement collection setup; (**b**) Test vehicle.

As an initial test case, range profile data of a mid-size sedan are collected. Figure 1a illustrates the measurement scenario and the inset figure shows the measurement collection setup that includes the horn and P410 radar connected to a laptop via USB. Figure 1b shows a photo of the measured vehicle. The vehicle is driven on a straight road with the radar positioned along the roadside. In this test case, two additional P410s with omnidirectional antennas are placed on the roof of the vehicle and at the location of the radar. They are used in the ranging radio mode to simultaneously collect ground truth position data as the vehicle drives by. After validation of the test case, the measurement setup (without the ranging radios) is moved to the side of a road to collect range profile data of traffic in a real-world scenario.

## 3. Motion Compensation and Image Formation Algorithms for Vehicles

The *k*-space imaging algorithm is used to generate the wide-angle ISAR imagery [1]. This is done through a 2-D inverse Fourier transform of the *k*-space data, as shown in the following formulation:

(1) $$\mathrm{Image}\left(x,y\right)=\iint E^{s}\left(k_{x}{,k}_{y}\right)e^{{\mathrm{jk}}_{x}x}e^{{\mathrm{jk}}_{y}y}{\mathrm{dk}}_{x}{\mathrm{dk}}_{y},$$

(2) $$\mathrm{where}\;\{\begin{array}{c} k_{x}=\frac{4\pi f}{c}\cos\phi \\ k_{y}=\frac{4\pi f}{c}\sin\phi \end{array},$$

and where x is the down-range, y is the cross-range, f is the frequency, ϕ is the aspect angle, $E^{s}$ is the backscattered field as a function of $k_{x}$ and $k_{y}$, and c is the speed of light.

Note that the *k*-space imaging formulation described above is for the standard turntable ISAR processing. Thus, the translational motion of the vehicle must be removed through motion compensation, retaining only the rotational motion. Due to the lack of persistent scatterers over the wide angular aperture, conventional narrow-angle ISAR focusing techniques are not applicable since they usually assume the availability of such scatterers.

We first use the radar cross section (RCS) centroid of the range profiles to form a coarse estimate of the vehicle range at each instance in time. The coarse estimate is then fitted to the following third-order motion model using least squares:

(3) $$r\left(t\right)=\sqrt{r_{\min}^{2}+{\left(a_{1}{t+a}_{2}t^{2}{+a}_{3}t^{3}\right)}^{2}}$$

Once the motion parameters ($r_{\min}$, $a_{1}$, $a_{2}$, and $a_{3}$) are found, we carry out the coarse motion compensation by multiplying the raw data in the (dwell time)-(real frequency) domain, $E^{s}(t,f)$, obtained through a 1-D fast Fourier transform in range, by a correction phase term:

(4) $$E_{\mathrm{aligned}}^{s}{(t,f)=E}^{s}{(t,f)e}^{+j\frac{4\pi f}{c}r(t)}.$$

Here, r(t) corresponds to the estimated range to the target center in each instance in dwell time. The measurement angle (ϕ) can found from r and $r_{\min}$ through the geometry in Figure 1a. The data are then polar-reformatted onto a uniform $k_{x}-k_{y}$ grid using a bilinear interpolation scheme. A 2-D inverse fast Fourier transform (IFFT) is then applied over the entire *k*-space to generate the wide-angle ISAR image.

Lastly, fine motion compensation is performed to obtain a focused image through a *p*-norm minimization. Using the motion model in Equation (2), a local optimizer is used to search for the motion parameters that minimize the *p*-norm of the image magnitude. The cost function is defined as:

(5) $$J={\left(\underset{\mathrm{pixels}}{\sum }{\left|A\right|}^{p}\right)}^{1/p}$$

where A is the magnitude of the resulting ISAR image at each pixel. Typically, 0≤p<1 is used to measure image sparsity [35], and p = 0.8 is chosen here. The MATLAB *fminsearch* optimizer is applied to search for the motion parameters ($a_{1}$, $a_{2}$, and $a_{3}$) that minimize the *p*-norm of the image magnitude. It is important to point out that the *p*-norm minimization contains many local minima, and thus a good initial guess of the motion parameters from the coarse motion compensation step is critical.

## 4. Vehicle Measurement Results

**Figure 2:**
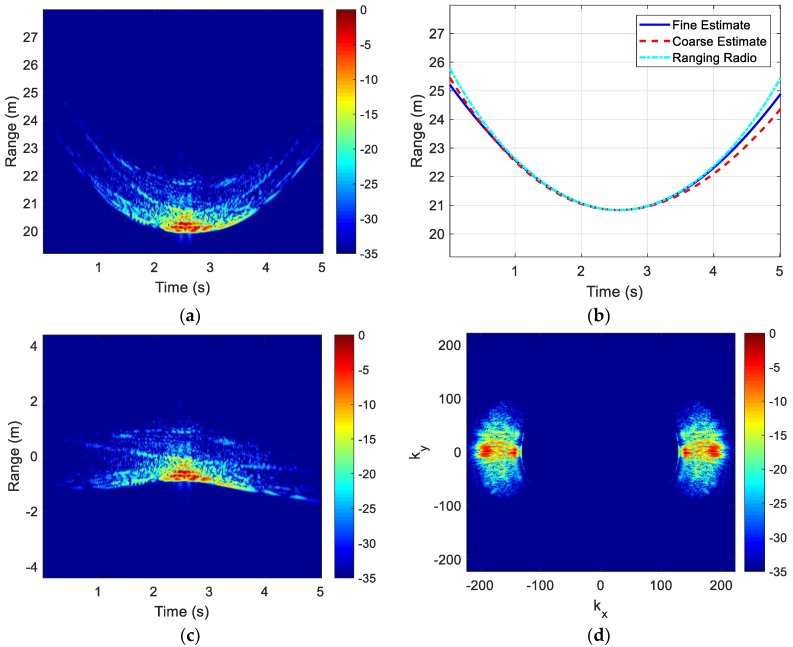
Vehicle measurement processing. (**a**) Raw range profiles; (**b**) Range alignment curves; (**c**) Aligned range profiles; (**d**) *k*-space data.

We start with the results for the test case where an instrumented sedan is driven at approximately 10 mph across the scene. Figure 2a shows the collected raw range profiles vs time. Significant range migration is observed due to the translational motion of the vehicle. The dashed red curve in Figure 2b shows the coarse range estimate based on the RCS centroid, after being fitted to the motion model. It can be compared to the dashed cyan curve, which shows the range estimate based on the ranging radio. Figure 2c shows the range profiles after coarse alignment to the RCS centroid. The data are then polar-reformatted onto a uniform $k_{x}-k_{y}$ grid. The *k*-space is only partially filled due to the limited bandwidth and measured angles. By assuming vehicle symmetry, the right half of the *k*-space can be mirrored to the left half. The resulting *k*-space data are shown in Figure 2d. A Hamming window is applied to the data in the ϕ-dimension to lower cross-range sidelobes. No windowing is applied in the frequency dimension since the frequency content of the radar pulse is already strongly tapered at the two band edges [18].

**Figure 3:**
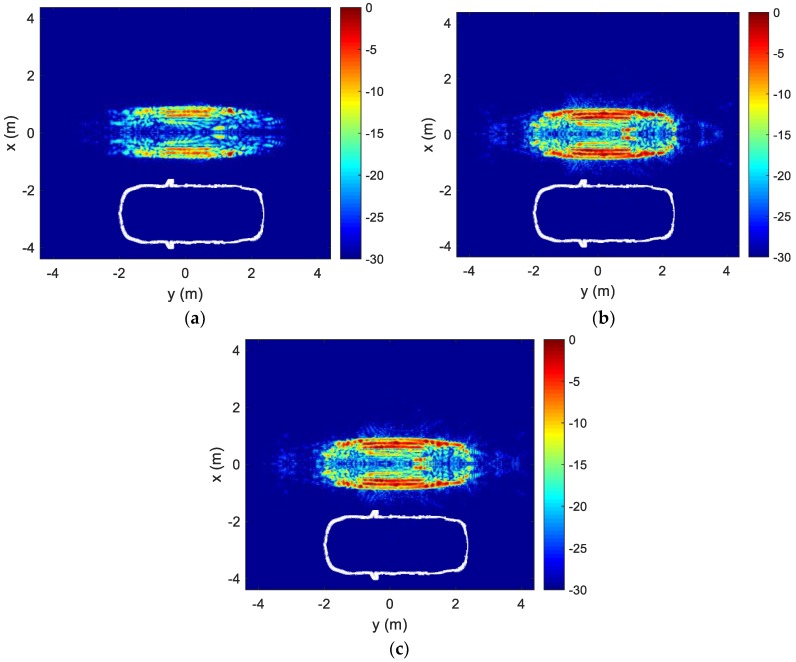
Vehicle ISAR images: (**a**) After coarse motion compensation; (**b**) After fine motion compensation; (**c**) After motion compensation using ranging radio data.

The resulting image, after coarse motion compensation, is shown in Figure 3a. The 2-D ISAR image in this figure (as well as all subsequent images) represents the relative RCS level in decibel. In addition, a white geometrical outline of the top-view of the vehicle is provided below the image for reference. An outline of the vehicle can be discerned in the ISAR image. The image after fine motion compensation is shown in Figure 3b. The solid blue curve in Figure 2b shows the corresponding range track after the *p*-norm minimization. The image in Figure 3b is now well focused. Note the red spot on the roof of the vehicle at y = 1 and slightly below x = 0. This is due to two front-facing, side-by-side corner reflectors that are intentionally placed on the roof of the vehicle. The focusing of the composite corner reflector in the final image further validates the result. Since we have mirrored the data in *k*-space, the spot also appears on the x≥0 side of the vehicle. The estimated down-range resolution of the image is 10 cm, and the estimated cross-range resolution of the image is 5 cm. Finally, as a reference for comparison, the image generated using the ranging radio data (without the *p*-norm minimization) is shown in Figure 3c. The ISAR image in Figure 3b after the blind motion compensation is on-par, if not better focused, than that of using the cooperative ranging radio data in Figure 3c. Based on the image in Figure 3b, the length and width of the vehicle are estimated to be 4.68 m by 1.82 m. They are close to the actual dimensions of the vehicle, which is 4.80 m by 1.83 m.

**Figure 4:**
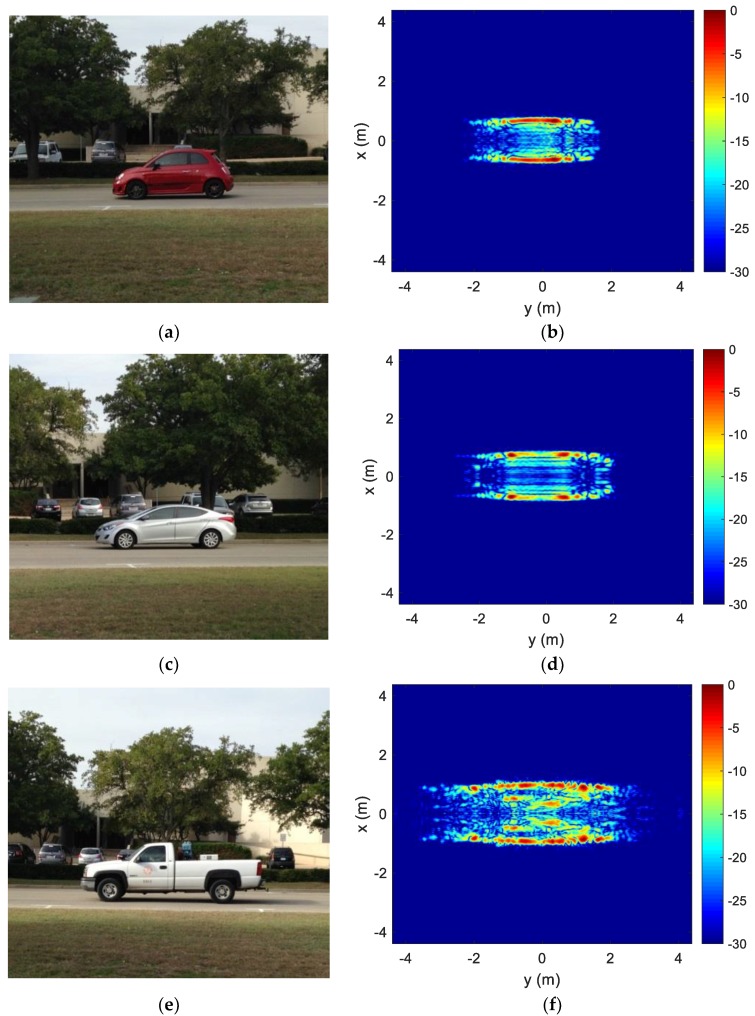
Wide-angle ISAR imaging of different vehicles. (**a**) Subcompact photo; (**b**) Subcompact ISAR image; (**c**) Mid-size sedan photo; (**d**) Mid-size sedan ISAR image; (**e**) Large truck photo; (**f**) Large truck ISAR image.

Next, vehicles are imaged in a real-world scenario. We place our measurement setup on the side of a public road on the University of Texas at Austin J. J. Pickle Research Campus in Austin, TX, and observe vehicles as they drive by. We present the resulting images of three different classes of vehicles: Subcompact, mid-size sedan, and large truck. Figure 4 shows a photo of each vehicle and their resulting ISAR image after blind motion compensation. Contrary to the test case presented earlier, these cases are in-situ and fully uncooperative scenarios, as we exert no control over the speed and acceleration of the imaged vehicles. Nonetheless, the image quality remains almost on-par with that from the test case. The images are well focused and reveal a clear distinction between the different-sized vehicles in both the length and width dimensions. Based on the images, the lengths and widths of the three vehicles are estimated to be 3.70 m by 1.60 m, 4.20 m by 1.62 m, and 5.45 m by 1.85 m, respectively.

While we have shown that it is possible to generate a focused ISAR image for moving vehicles on the road under the wide-angle scenario, two comments are in order. First, it is noted that target features in the front and rear of the vehicles are lacking. Observing the vehicles over a wider angular swath could reveal more of these features. In the collected data set, we are able to cover up to a 70° angular swath. This is limited largely by the beamwidth of the horn antenna. Consequently, there will be a tradeoff between gathering more frontal features and SNR. Second, it is noted that the sides of the vehicle are slightly more curved than their geometrical shapes in all the acquired images. The effect is most prominent in the large truck in Figure 4f. This distortion is likely due to the near-field effect from the large flat side surface of the vehicles. Such image distortion may be compensated by using a near-field backprojection algorithm [36] or a near-field to far-field transform [37,38]. This topic is beyond the scope of the present study. Third, the collected data contain only one vehicle during the collection interval. If multiple vehicles are present in the scene, the target returns will need to be separated in the range and/or Doppler space before the image formation process.

## 5. Drone Imaging and Measurement Setup

**Figure 5:**
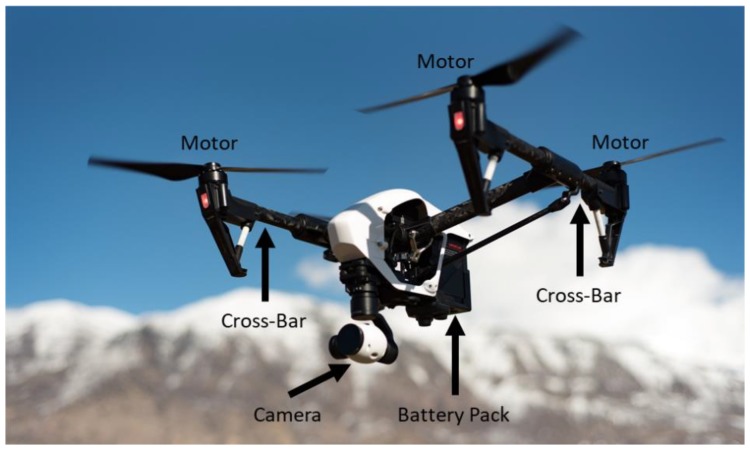
DJI Inspire 1 [48].

Small drones have been gaining popularity for uses in aerial photography, surveying, mapping, and package delivery. Their proliferation has also raised recent interest in their regulation and monitoring [39,40,41,42]. A potential way to detect and identify drones is to use ground-based radar. Analogous to vehicles, it should be possible to generate an ISAR image of a small drone using radar as the drone flies across the measurement scene. However, there are several additional challenges that need to be overcome in order to obtain a wide-angle ISAR image of a small drone: (i) The small size and low reflectivity of the plastic body may result in a very low radar cross section; (ii) the spinning blades of the drone may result in significant dynamic signature features similar to other rotorcraft [43,44,45]; and (iii) the potentially unsteady flight of a typical drone may complicate motion compensation, making image formation more challenging. We have addressed the first two challenges by conducting laboratory measurements of several small consumer drones and examining their ISAR images in Reference [46]. ISAR measurement of two other drones was reported in Reference [47]. Here, we investigate the last challenge through in-situ measurements. The target is a DJI Inspire 1 quadcopter [48], and its key structural features are labeled in Figure 5. Results from the most relevant data are shown here. Readers are referred to Reference [49] for additional drones and flight paths.

**Figure 6:**
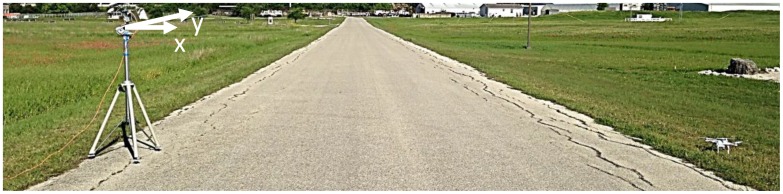
In-flight drone measurement setup.

In-flight measurements of the quadcopter drone are taken using a more recently released version of the Time Domain radar, the P440 [13], placed on the ground. A single dual-ridged horn (TDK HORN-0118, 1–18 GHz) is used for both transmit and receive through the use of a circulator. Vertical polarization is used on transmit and receive. Range profiles are collected as the Inspire 1 is flown along an approximate straight path, at about 9 m away from the radar, across the measurement scene. The flight is held at a constant altitude of roughly 1.8 m above the ground. The sampling rate for consecutive range profiles is set to 100 Hz. Figure 6 shows the measurement setup with the measurement equipment on the left and a quadcopter drone on the right. Table 2 summarizes the measurement parameters.

## 6. Motion Compensation and Image Formation Algorithms for Drones

Contrary to vehicles that move along a straight road, small drones can have flight paths that are not constrained to a straight line. In addition, the size of these small drones typically spans only a few range cells in down-range. Therefore, the motion compensation algorithm described in Section 3 must be modified to process the radar data of a small drone over a wide angle to achieve a well-focused image. The modified algorithm is described below.

First, we propose a more general motion model in both *x* and *y* dimensions:

(6) $$x\left(t\right)=\overset{N}{\underset{n=0}{\sum }}a_{n}t^{n},\;y\left(t\right)=\overset{M}{\underset{m=0}{\sum }}b_{m}t^{m},$$

where the radar is assumed to be located at the origin. Based on this model, the range and Doppler expressions are:

(7) $$\vec{r}=x\hat{x}+y\hat{y},\;r=\sqrt{x^{2}{+y}^{2}}$$

(8) $$\vec{v}=\dot{x}\hat{x}+\dot{y}\hat{y}=\overset{N}{\underset{n=1}{\sum }}{\mathrm{na}}_{n}t^{n-1}\hat{x}+\overset{M}{\underset{m=1}{\sum }}{\mathrm{mb}}_{m}t^{m-1}\hat{y},\;f_{D}=-\frac{2f}{c}\vec{v}\cdot \frac{\vec{r}}{r}$$

Therefore, once the motion parameters $\left\{a_{n}\right\}$ and $\left\{b_{m}\right\}$ are found, the range r and Doppler $f_{D}$ can be readily determined.

Next, we carry out coarse motion compensation using both range and Doppler information from the measurement data. This is an extension of the range-only coarse motion compensation used for vehicle imaging. In addition to the range centroid extracted from the range profile data, we also extract the Doppler centroid information. This is done by applying the short-time Fourier transform to the frequency response over dwell time at a chosen frequency, using a Hamming window of 40 ms, to yield the spectrogram or Doppler profile. This provides the instantaneous Doppler frequency of the target over time. A Doppler centroid is then extracted at each time instant.

We then carry out an optimization to determine the unknown motion parameters $\left\{a_{n}\right\}$ and $\left\{b_{m}\right\}$ by simultaneously matching to the extracted range and Doppler trajectories of the measurement. The cost function in the minimization is defined as:

(9) $$J=\underset{t}{\sum }\|r\left(t\right)-r_{\mathrm{meas}}\left(t\right){\|}^{2}+\alpha \|f_{D}\left(t\right)-f_{D,\mathrm{meas}}\left(t\right){\|}^{2}$$

In the cost function, α controls the relative weight between Doppler and range errors. The MATLAB routine *fminunc* is used in the optimization. In addition, it is found that a regularization term, which is proportional to the variance of the velocity from the approximate measured velocity of the drone, should be added to the total cost to arrive at a stable solution. Once the motion parameters are found, we carry out the motion compensation using Equation (4).

After coarse motion compensation, the data can be placed into *k*-space by converting dwell time to the corresponding aspect angle as follows:

(10) $$\phi {(t)=\sin}^{-1}(\hat{v}\cdot \hat{r})$$

where v^ and r^ are unit vectors in the velocity and down-range directions, respectively. Via the standard polar-to-rectangular reformatting operation and a 2-D inverse FFT, we arrive at the ISAR image of the target.

Lastly, we carry out the same fine motion compensation based on *p*-norm minimization that was described in the vehicle imaging. The optimal $\left\{a_{n}\right\}$ and $\left\{b_{m}\right\}$ are searched for to arrive at a focused image.

## 7. Drone Measurement Results

**Figure 7:**
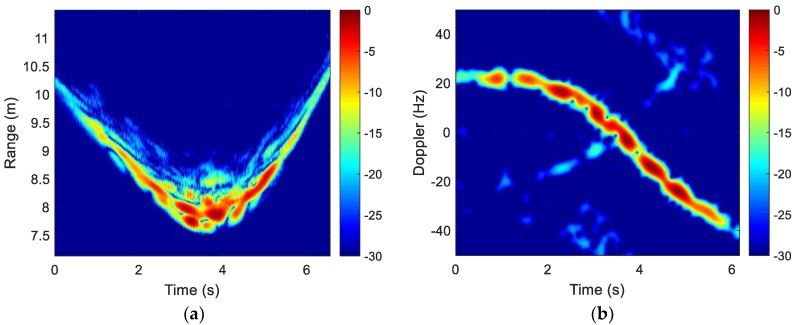
Drone in-flight raw data. (**a**) Range profiles; (**b**) Doppler profiles.

One set of collected range profiles are shown in Figure 7a. The corresponding Doppler profile at 4.20 GHz is shown in Figure 7b. Again, motion compensation needs to be applied to remove the translational motion.

**Figure 8:**
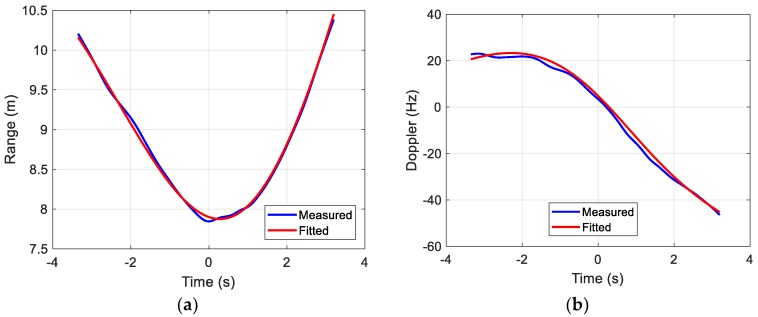
Drone in-flight range/Doppler curve fitting. (**a**) Range vs. time; (**b**) Doppler vs. time.

The RCS centroids in range and Doppler are first extracted from the range profiles and Doppler profiles. The cost function in Equation (9) is then minimized to generate the fitted curves. Figure 8a shows a comparison of the fitted curve to the extracted curve for range. Figure 8b shows a comparison of the fitted curve to the extracted curve for Doppler. To generate these fitted curves, the order of the model is set to *N* = *M* = 3 and α is set to 0.001 $m^{2}/{\mathrm{Hz}}^{2}$ in Equation (9).

**Figure 9:**
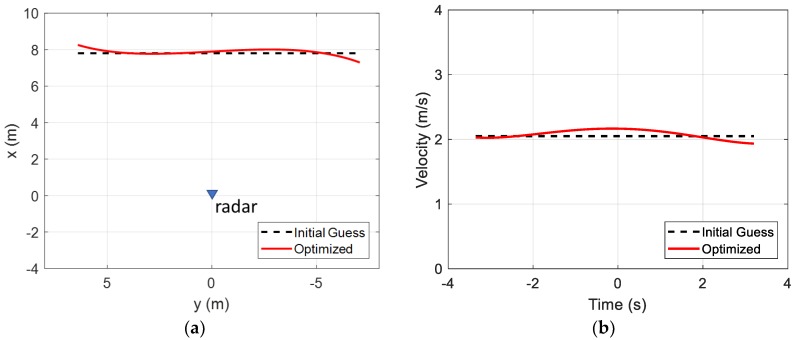
Drone in-flight fitted position and velocity. (**a**) Position; (**b**) Velocity.

The corresponding drone position in x and y, using the fitted data, is shown in Figure 9a. Similarly, the corresponding drone velocity is shown in Figure 9b. The initial guess assumes a straight flight path with a constant velocity. It is clear that the fitted drone flight deviates from such astraight-line, constant-velocity flight.

**Figure 10:**
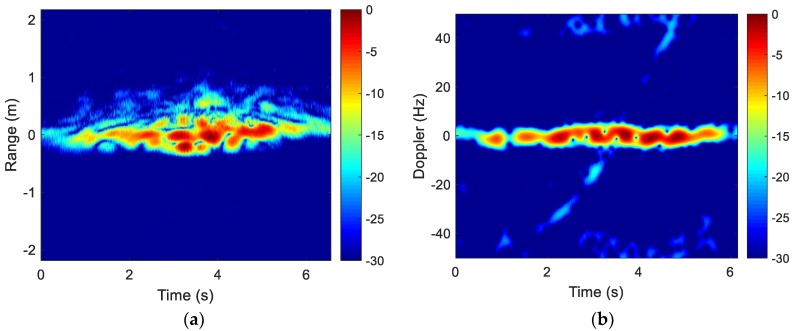
Drone motion compensated data. (**a**) Aligned range profiles; (**b**) Aligned Doppler profiles.

After using Equation (4) to align the collected data based on the fitted curve shown in Figure 8a, we obtain the aligned range and Doppler profiles shown in Figure 10. The translational motion has been largely removed.

**Figure 11:**
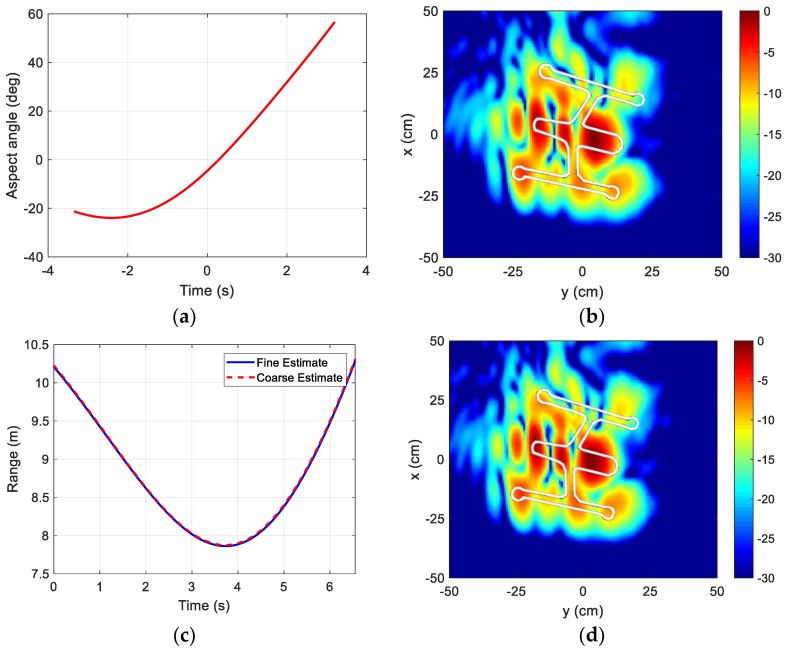
Drone ISAR image formation. (**a**) Drone aspect angle vs. time; (**b**) Coarse motion compensation image; (**c**) Coarse vs. fine estimate; (**d**) Fine motion compensation image.

Finally, the data are placed into the *k*-space by converting dwell time to the corresponding aspect angle using Equation (10). The resulting aspect angle vs. time is shown in Figure 11a. The image after coarse motion compensation is shown in Figure 11b. The geometrical outline of the drone in white is overlaid onto the ISAR image for comparison. The outline of the drone body can be clearly seen, with the strongest return arising from the battery pack and camera around the central fuselage. Next, a fine motion compensation using *p*-norm minimization is carried out. Figure 11c compares the range trajectories before and after *p*-norm minimization. Figure 11d shows the final image after fine motion compensation. We do not observe a large difference in image quality before and after the fine motion compensation. The cost function in Equation (4) decreases only slightly from the initial value of 273 to 269 after the minimization. The estimated down-range resolution of each image is 10 cm, and the estimated cross-range resolution of each image is 5 cm. The outline of the drone body can be clearly seen. By correlating the hot spots in the ISAR image with the prominent geometrical features of the drone, the strongest returns appear to arise from the battery pack and camera around the central fuselage.

**Figure 12:**
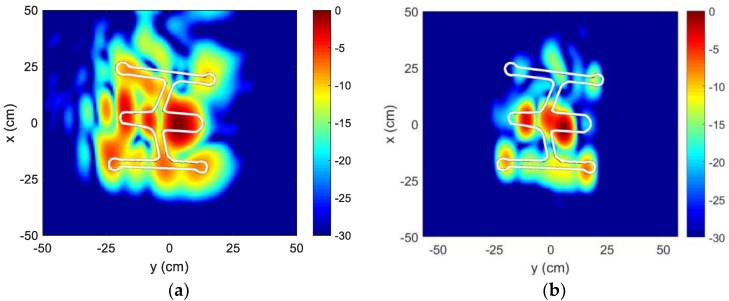
Reduced-aperture ISAR image. (**a**) In-flight; (**b**) Laboratory.

It is worth noting that even after fine motion compensation, there are still some out-of-body features in both x and y dimensions. The former, which extends beyond the drone (in down-range), is likely due to multiple scattering effects. The latter, along the cross-range dimension, may be due to the drone flight not fully following the motion model over the 78° angular swath. In Figure 12a, by narrowing the angular swath to 55° (−22° to +33° in this case), we can reduce the amount of out-of-body features along the y-dimension. For comparison, the image based on laboratory turntable data for another Inspire 1 drone [46], using a 55° angular swath with a signal bandwidth from 3.1 to 5.3 GHz, is shown in Figure 12b. By comparing Figure 12a,b, we see that the main body features are present in both images: Drone battery pack, camera, and cross-bars. The 4 motors that sit at the end of the cross-bars are also visible. It is worth noting that the target image in Figure 12b looks smaller than that in Figure 12a. This could be because the drone has a larger geometrical footprint when in flight than when it is in landing mode (during laboratory measurement). The white geometrical outline is of the drone in landing mode.

## 8. Conclusions

In this paper, we investigated the wide-angle, ultra-wideband ISAR imaging of moving vehicles and an in-flight drone. We developed measurement methodologies, collected in-situ data using UWB transceivers from Time Domain, and implemented motion compensation and image formation algorithms to form high-resolution radar images.

For moving vehicle imaging, it was shown that a UWB radar placed along roadside can be used to acquire high-resolution, 2-D vehicle imagery. A coarse motion compensation via range centroid alignment, followed by a *p*-norm minimization of the resulting image, was used to generate a focused image over a 70° aperture. Results of the blind motion compensation were checked against a test case, where the vehicle was instrumented with a ranging radio. Roadside collection was also conducted in a non-cooperative scenario. The resulting images were also well focused and corresponded closely to the physical dimensions of the vehicles.

For in-flight drone imaging, motion compensation was more challenging due to the less constrained flight path, more chaotic target motions, and the small size of the target. In the motion compensation process, we used a more general motion model to account for the non-linear flight path, aligned both the range and Doppler centroids of the target, and carried out *p*-norm minimization. It was shown that a high-resolution image could still be captured for the drone under test. Strong scattering from the non-plastic parts on the drone including the battery pack, motors, and carbon fiber frames could be clearly identified in the resulting image. The image also compared favorably against that generated from laboratory turntable measurement.

Overall, we have shown that wide-angle, UWB ISAR imaging is feasible on moving vehicles and in-flight drones. It is possible to achieve a cross-range resolution that is on par with the down-range resolution, provided that the motion compensation issue is properly addressed. The resulting images reveal important 2-D features on the target. A potential follow-on study would entail collecting data for a large set of targets and using the acquired high-resolution images for radar target recognition.

## Figures and Tables

**Table 1:** Vehicle imaging measurement parameters.

Parameter	Value
Equivalent frequency bandwidth	3.1–5.3 GHz
Transmit power	0.7 dBm
Pulse repetition frequency	10 MHz
Coherent pulse integration	1024 pulses
Closest range	~20 m
Antenna beamwidth	~70°
Antenna polarization	vertical (Tx), vertical (Rx)

**Table 2:** Drone imaging measurement parameters.

Parameter	Value
Equivalent frequency bandwidth	3.1–5.3 GHz
Transmit power	−3 dBm
Pulse repetition frequency	10 MHz
Coherent pulse integration	2048 pulses
Closest range	~20 m
Antenna beamwidth	~70°
Antenna polarization	vertical (Tx), vertical (Rx)
